# Efficacy of superselective transcatheter arterial embolization for intractable postpartum hemorrhage due to genital tract trauma after vaginal delivery

**DOI:** 10.1007/s10140-021-01971-w

**Published:** 2021-07-24

**Authors:** Koji Sasaki, Takuya Okada, Masato Yamaguchi, Mostafa Ahmed, Tomoyuki Gentsu, Eisuke Ueshima, Keitaro Sofue, Kenji Tanimura, Hideto Yamada, Koji Sugimoto, Takamichi Murakami

**Affiliations:** 1 Department of Radiology, Kobe University Graduate School of Medicine, 7-5-2, Kusunoki-cho, Chuo-ku, Kobe, Japan; 2 Department of Obstetrics and Gynaecology, Kobe University Graduate School of Medicine, 7-5-2, Kusunoki-cho, Chuo-ku, Kobe, Japan

**Keywords:** Selective transcatheter arterial embolization, Postpartum hemorrhage, Genital tract trauma, Pre-procedural contrast-enhanced computed tomography

## Abstract

**Purpose:**

To evaluate the efficacy of superselective transcatheter arterial embolization (TAE) for intractable postpartum hemorrhage (PPH) due to genital tract trauma (GTT) after vaginal delivery.

**Methods:**

We evaluated 27 patients who underwent TAE for intractable PPH due to GTT after vaginal delivery at our institution between January 2008 and December 2020. Patients were divided into two groups according to TAE procedure; TAE performed as close as possible to the bleeding point, at least more peripherally than the second branch of the anterior division of the internal iliac artery, was defined as superselective TAE (S-TAE). TAE performed from the proximal segment of the internal iliac artery was defined as proximal TAE (P-TAE). Patient characteristics, pre-procedural contrast-enhanced computed tomography (CE-CT), procedure details, technical/clinical success, and complications were evaluated separately for the S-TAE and P-TAE groups.

**Results:**

The combined technical/clinical success rate was 92%. No major procedure-related complications were seen (mean follow-up: 6.12 ± 3.93 days). The combined technical/clinical success rate of S-TAE was 100% and of P-TAE was 67% (*p* = 0.04). S-TAE was performed more frequently in patients with pre-procedural CE-CT (*p* = 0.01) and use of permanent embolic materials (*p* = 0.003).

**Conclusion:**

S-TAE is safe and effective for intractable PPH due to GTT. Pre-procedural CE-CT may be useful for detecting the culprit artery and be helpful in performing S-TAE.

## Introduction

Postpartum hemorrhage (PPH) remains the leading cause of maternal death worldwide [1, 2]. PPH is defined by the American College of Obstetrics and Gynecology as cumulative blood loss greater than 1000 mL with signs and symptoms of hypovolemia within 24 h of the birth process, regardless of the route of delivery [3]. Multiple conditions cause PPH, and the well-known four-T mnemonic can be used to identify and address the four most common causes of PPH: uterine atony (tone); laceration, hematoma, inversion (trauma); retained tissue or placenta accreta (tissue); and coagulopathy (thrombin) [4]. Among them, the most common is uterine atony due to inadequate contraction of the uterine muscle, accounting for approximately 70% of all PPH cases, followed by trauma, including genital tract trauma (GTT), which accounts for approximately 20% of cases [4].

Bilateral uterine artery embolization (UAE) is an established treatment for uterine causes of intractable PPH, including uterine atony, retained tissue, or placenta accreta [5–7]. However, intractable PPH due to GTT, where hemostasis by sutures is difficult to achieve, does not require bilateral UAE but rather transcatheter arterial embolization (TAE) of the bleeding point. Several studies have demonstrated the high effectiveness of TAE for intractable PPH due to GTT [8–10]. However, some reports have suggested that the blood vessels that cause bleeding due to GTT are diverse, and there is a risk of re-bleeding from anastomotic branches [11, 12]. In addition, other reports do not strictly distinguish between bleeding due to uterine atony and PPH due to GTT; thus, these conditions are often confused.

In our institute, to achieve complete hemostasis and reduce the rate of re-bleeding from anastomotic branches, superselective TAE has recently been employed; that is, the target vessel is embolized as close as possible to the bleeding point. However, no studies to date have examined in detail the relationship between embolization methods and TAE outcomes.

The purpose of this study was to evaluate the efficacy of superselective TAE for intractable PPH due to GTT after vaginal delivery.

## Materials and methods

### Patient selection

Patients who underwent TAE for intractable PPH due to GTT after vaginal delivery, in which hemostasis was difficult to achieve by suturing, at our institution between January 2008 and December 2020 were included in the study. Patients scheduled for surgery prior to TAE were excluded.

### Pre-procedural contrast-enhanced computed tomography

Pre-procedural abdominal contrast-enhanced computed tomography (CE-CT) was performed at the discretion of the physician to confirm active bleeding using a 16-channel multidetector-row CT scanner (Brilliance-16; Philips Medical Systems, Best, Netherlands), a 64-channel (Aquilion ONE or 64; Cannon Medical Systems, Otawara, Japan), or a 192-channel multidetector-row CT scanner (SOMATOM Force; Siemens Healthcare, Forchheim, Germany). An unenhanced whole abdominal scan was obtained in transverse section. Dual-phasic CE-CT was performed 30‒45 s and 100‒120 s after the start of intravenous administration of contrast material with 5 mm- and 0.5‒1.0-mm slice thickness. Iodinated non-ionic contrast material was injected at a dose of 510 mgI/kg of body weight with fixed injection duration of 30 s.

### Embolization procedure

**Fig. 1:**
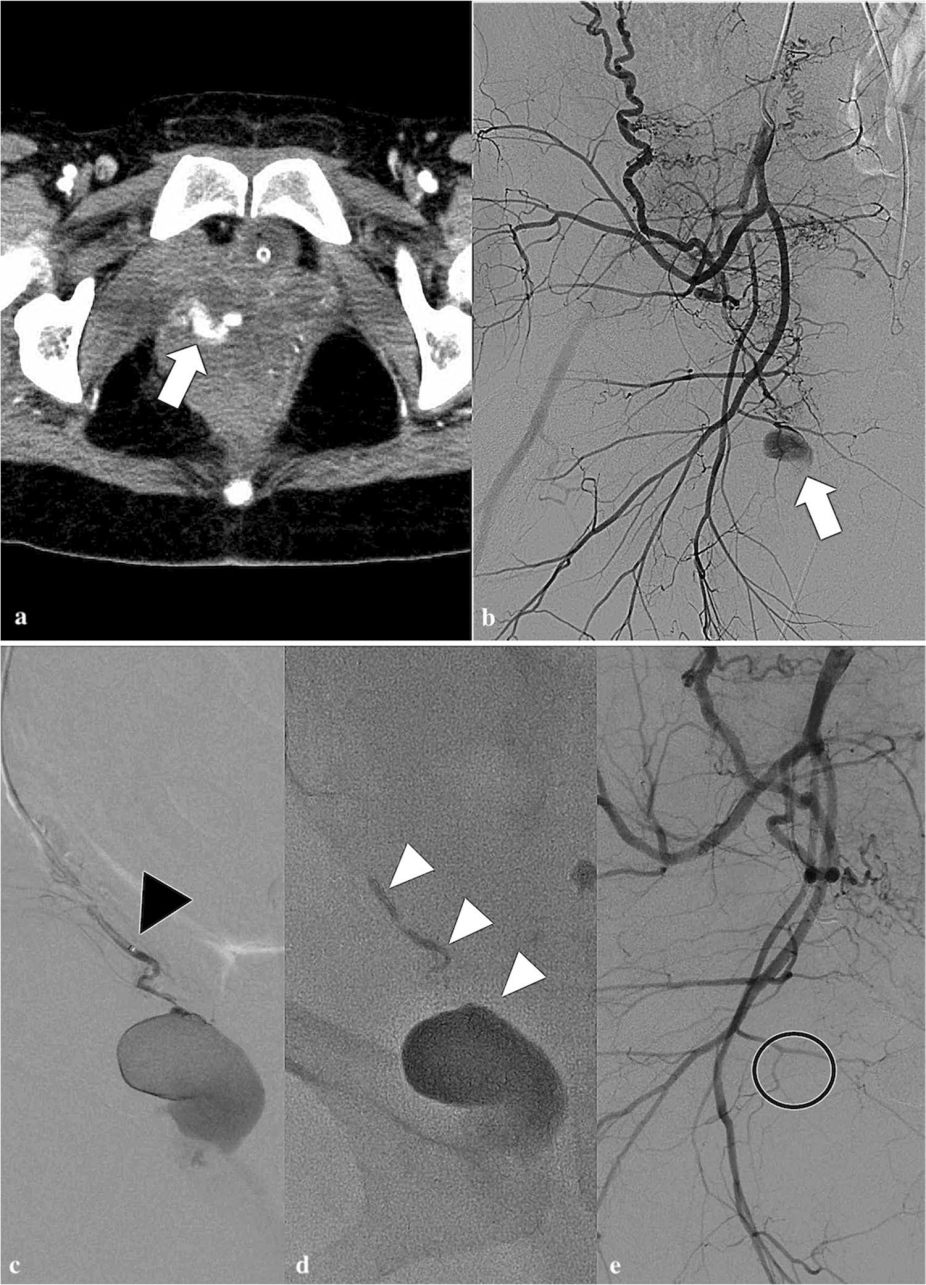
A typical case of superselective transcatheter arterial embolization. A 32-year-old woman with right vaginal hematoma presenting with postpartum hemorrhage after vaginal delivery. **a** Pre-procedural contrast-enhanced computed tomography shows extravasation of contrast medium in the right vaginal wall (white arrow). **b** Right internal iliac arteriography demonstrates a pseudoaneurysm from the right vaginal artery (white arrow). **c** Superselective angiography from the right vaginal artery; the black arrowhead points to the tip of the microcatheter. **d** The right vaginal artery was embolized with 33% NBCA-lipiodol mixture (white arrow heads). **e** The right internal iliac arteriography after embolization demonstrates disappearance of the pseudoaneurysm (black circle)

Informed consent was obtained from all patients or patients’ families prior to the procedure. Under local anesthesia, 4- or 5-French (F) sheath was introduced via the left or right common femoral artery. Pelvic angiography was performed to identify the bleeding point and the target vessels. If the bleeding point could not be identified on angiography, the target vessel was selected based on the CE-CT findings or the location of hematoma. A microcatheter (1.7–2.3-F) was inserted into the target vessel, and the tip of the microcatheter was placed as close as possible to the bleeding point. We performed embolization as close as possible to the bleeding point, at least more peripherally than the second branch of the anterior division of the internal iliac artery. The target vessel was embolized using a mixture of n-butyl-2-cyanoacrylate (NBCA, Histoacryl, B. Braun, Melsungen, Germany) and iodized oil (Lipiodol; Guerbet, Aulnay-sous-Bois, France). Gelatin sponge particles (GS, Serescue, Nippon Kayaku, Tokyo, Japan), which were cut into 0.5–1.0 mm-cubes using a scalpel and scissors, were used sequentially as needed. The selection of embolization materials and the range of NBCA/Lipiodol ratio (20–50%) were determined by the operator. If the catheter could not be advanced to the target branch, we performed embolization from the proximal site of the parent artery (the first branch or the main trunk of the anterior division of the internal iliac artery in most cases) with GS particles. We defined the former embolization method as superselective TAE (S-TAE) (Fig. 1) and the latter as proximal TAE (P-TAE).

Digital subtraction angiography and gynecological examination were performed immediately after TAE to confirm the cessation of bleeding. When cessation of bleeding was not achieved, additional TAE was performed. The procedure was completed after confirming the cessation of bleeding on angiography and on speculum examination immediately after TAE. Bilateral UAE was performed for patients suspected of concurrent uterine atony.

### Evaluation factors and definitions

We reviewed patients’ medical records and collected the following data: maternal characteristics, comorbidities, hematoma location, mean hemoglobin value, shock index (heart rate (HR)/systolic blood pressure (SBP)), presence of coagulopathy, whether pre-procedural CE-CT obtained or not, angiographic findings, TAE procedure, technical and clinical outcomes of TAE, and complications related to the procedure. Coagulopathy was defined as a platelet count of less than 50,000/mm^3^, an international normalized ratio greater than 1.5, or serum fibrinogen concentration less than 150 mg/dL. Two interventional radiologists with 17 and 12 years’ experience independently reviewed the pre-procedural CE-CT images and angiographic findings/records. All pre-procedural CE-CT and angiographic images were reviewed on a dedicated picture archiving and communication viewer. For each patient, the presence of active bleeding, hematoma location, target vessels, procedure time, and embolic materials were recorded. The procedure time was defined as the time between initial pelvic arteriography and post-embolization pelvic arteriography. We judged by consensus whether the procedure was S-TAE or P-TAE, and whether there was technical success or failure. Technical success was defined as the cessation of bleeding on angiography and on speculum examination by an obstetrician immediately after TAE. Clinical success was defined as the cessation of bleeding without repeated TAE and/or surgery during the hospital stay. Complications were categorized as major or minor according to the guidelines of the Society of Interventional Radiology Standards of Practice Committee [13]. Major complications were defined as those requiring major therapy, necessitating an unplanned increase in the level of care or prolonged hospitalization (more than 48), or resulting in permanent adverse sequelae or patient death. We evaluated all these data separately for the S-TAE and P-TAE groups.

### Statistical analysis

Continuous variables were analyzed using Mann–Whitney’s *U* test, and qualitative variables were analyzed using Fisher’s exact test to compare the two groups. All *p*-values less than 0.05 were considered statistically significant. All statistical analyses were performed with EZR software (Saitama Medical Center, Jichii Medical University, Saitama, Japan), which is a graphical user interface for R (The R Foundation for Statistical Computing, Vienna, Austria). More precisely, it is a modified version of R commander designed to add statistical functions frequently used in biostatistics [14].

## Results

### Patients’ characteristics

Between January 2008 and December 2020, 28 patients underwent TAE for intractable PPH due to GTT after vaginal delivery. One patient scheduled for surgery for removal of a large hematoma prior to TAE was excluded. Consequently, 27 patients (mean age, 31.8 years; range, 20–42 years) were included in this study.

The detailed characteristics of all patients and for each group classified by embolization method are shown in Table 1. There was no difference in patients’ backgrounds between the S-TAE and P-TAE groups.

**Table 1:** Details of patient characteristics for each embolization method

Characteristics	Overall (*n* = 27)	S-TAE group (*n* = 21)	P-TAE group (*n* = 6)	*p*-value
Age (years)	31.8 ± 5.7	30.9 ± 5.7	35.8 ± 4.0	*.12*
Maternal characteristics				*.52*
Primiparity	17	12	5	
Presentation to the hospital				*.40*
Referred patient	25	20	5	
Inpatient	2	1	1	
Timing of postpartum hemorrhage				*.11*
Within 24 h of delivery	24	20	4	
Laboratory data				*.23*
Hemoglobin (g/dl)	8.6 ± 2.1	8.33 ± 2.0	9.48 ± 2.2	
Shock index; HR (bpm)/SBP (mmHg)				*.55*
More than 1.0	5	5	0	
Massive transfusion				*.66*
> 10 RBC units	12	10	2	
Coagulopathy				*1.000*
Fibrinogen < 150 mg/dl	5	4	1	
Pre-procedural CE-CT				.011*
Obtained	21	19	2	
Location of hematoma				*1.000*
Vulvar hematoma	4	3	1	
Vaginal hematoma	23	18	5	
Number of bleeding arteries				*.28*
Multi	7	4	3	
Embolic materials				.0031*
GS only	12	6	6	
NBCA or coils ± GS	15	15	0	
Combined use of UAE	3	1	2	*.11*
Procedure time (min)	52.0 ± 33.1	48.7 ± 20.9	66.0 ± 56.2	*.95*
Hospital stay (days)	6.1 ± 3.8	5.6 ± 3.6	7.8 ± 4.4	*.27*

Data are presented as numbers or mean ± standard deviation. *p < .05

*S-TAE*, super selective transcatheter arterial embolization; *P-TAE*, proximal transcatheter arterial embolization; *HR*, heart rate; *SBP*, systolic blood pressure; *RBC*, red blood cell; *CE-CT*, contrast-enhanced computed tomography; *GS*, gelatin sponge; *NBCA*, N-butyl-2-cyanoacrylate; *UAE*, uterine artery embolization

### Pre-procedural CE-CT and angiographic findings

Pre-procedural CE-CT was performed in 21 of the 27 patients to confirm the presence of active bleeding and determine the need for TAE. CE-CT was performed in 90% of the S-TAE group significantly more than in 33% of the P-TAE group (*p* = 0.01). We were able to identify active bleeding in all 21 patients who underwent pre-procedural CE-CT. However, in 5 patients, we were unable to identify active bleeding on the first pelvic angiography (PAG), and active bleeding was visualized by selective angiography based on CT findings. Among the six patients who did not undergo CE-CT, the first PAG identified active bleeding signs in five patients but not in one patient. However, active bleeding was visualized by selective angiography based on location of hematoma.

### Characteristics of TAE

A total of 34 bleeding vessels were detected in 27 patients (Table 2). Of these, 33 vessels could be embolized, whereas only one vessel could not be embolized because of spasm. Of the 27 patients, 20 (74%) had active bleeding from a single vessel, whereas 7 (26%) had active bleeding from multiple vessels. The vaginal artery was the most frequent artery that caused bleeding (*n* = 17, 50%), followed by the perineal artery (*n* = 8, 23%), internal pudendal artery (*n* = 4, 12%), obturator artery (*n* = 3, 9%), inferior mesenteric artery (*n* = 1, 3%), and inferior rectal artery (*n* = 1, 3%). GS was used as the sole embolic material in 12 (44%) and NBCA or coils ± GS in 15 patients (56%). NBCA was used as the main embolic material in 13/27 patients (48%). In the S-TAE group, permanent embolic materials such as NBCA and metallic coils were used significantly more than in the P-TAE group (*p* = 0.003) (Table 1). Concomitant bilateral UAE was performed in three patients (11%) suspected to have concurrent uterine atony.

**Table 2:** Blood vessels that caused bleeding

Arteries	Number (%)
Vaginal artery	17 (50%)
Perineal artery	8 (23%)
Internal pudendal artery	4 (12%)
Obturator artery	3 (9%)
Inferior mesenteric artery	1 (3%)
Inferior rectal artery	1 (3%)
Total	34 (100%)

### Outcomes of TAE

The technical success rate of TAE was 96% (26/27), and the clinical success rate was 96% (26/27). The combined technical and clinical success rate was 92% (25/27) (Table 3). The combined technical and clinical success rate of S-TAE was 100% (21/21), significant higher than of P-TAE was 67% (4/6) (*p* = 0.04). In one case of technical failure, hemostasis was achieved by vaginal packing. In one case of clinical failure in the P-TAE group, re-bleeding occurred the day after the first procedure; we performed S-TAE with NBCA at the second TAE and confirmed hemostasis. None of the patients required surgery for hemostasis. All patients were discharged or transferred back to the referring clinic without sequelae. No major complications related to the embolization were seen in any of the patients (follow-up: 6.12 ± 3.93 days, mean ± SD).

**Table 3:** Technical and clinical results of transcatheter arterial embolization (TAE) for each embolization method

	Overall (*n* = 27)	S-TAE group (*n* = 21)	P-TAE group (*n* = 6)	*p*-value
Technical success	26/27 (96%)	21/21 (100%)	5/6 (83%)	*0.22*
Clinical success	26/27 (96%)	21/21 (100%)	5/6 (83%)	*0.22*
Technical and clinical success	25/27 (92%)	21/21 (100%)	4/6 (67%)	0.04*

Data are presented as numbers or mean ± standard deviation. ^*^p < 0 .05

*S-TAE*, superselective transcatheter arterial embolization; *P-TAE*, proximal transcatheter arterial embolization

## Discussion

In the previous study focusing on TAE for intractable PPH due to GTT, the technical success rate was high at 98%, but the clinical success rate was slightly lower at 88% [8]. In our study, both the technical and clinical success rates were high at 96%. The combined technical and clinical success rates in this study were perfect at 100% in cases of S-TAE, and as low as 67% in cases of P-TAE. In the aforementioned study, the authors concluded that massive transfusion, hematoma location, and long hospital stay were related to failure of bleeding control [8]. However, the relationship between the detailed embolization method, such as the position of the microcatheter at the start of the embolization and TAE results was not clarified. There have been other case reports and small case series of GTT requiring re-TAE due to persistent bleeding via anastomosis from the inferior mesenteric artery after embolization of the internal iliac artery [11, 12]. Our findings indicated that S-TAE is effective with a high rate of complete hemostasis for intractable PPH due to GTT after vaginal delivery.

The pelvic artery has abundant collateral vessels, and multiple vessels contribute to the bleeding. Multiple target vessels were present in 24% of patients in our study and in 25% in a previous study [8]. There may also be potential collateral vessels that cannot be identified on imaging due to hematoma or vascular spasm. We speculate that embolization of the bleeding point with S-TAE may prevent re-bleeding from the collateral vessels and recanalization of the embolized vessel. The use of permanent embolic materials in many cases of S-TAE may also be related, although embolic materials were not related to the failure of bleeding control in the previous report [8]. We used NBCA for about half of the patients in this study, which is more frequent than previously reported [8–10]. Several studies have reported that TAE using NBCA was effective to prevent recanalization, particularly in cases of pseudoaneurysm or active bleeding with extravasation [15, 16]. We believe that these findings are also applicable to PPH due to GTT.

Pre-procedural CE-CT is another factor that contributed to our results. In this study, active bleeding, such as extravasation and pseudoaneurysms, was detected in all patients who underwent pre-procedural CE-CT; however, in five patients, the first pelvic angiography could not identify the active bleeding. This is consistent with the findings of other studies showing that CE-CT is more sensitive for identifying bleeding than non-selective digital subtraction angiography [17, 18]. In addition, S-TAE was performed significantly more frequently in patients who underwent pre-procedural CE-CT than in those who did not. Although there are many reports on the usefulness of CE-CT for identifying the presence of active bleeding before angiography under different circumstances (e.g., gastrointestinal bleeding, trauma) [19, 20], few studies have examined its role in obstetrical bleeding. This is because the most common cause of PPH is uterine atony. Intractable PPH from the uterus can be controlled by performing bilateral UAE, thus reducing the need for preoperative imaging. In the case of PPH due to GTT, various blood vessels (not only the uterine artery) can cause bleeding [8–10]. Pre-procedural CE-CT could be helpful in performing S-TAE and improving the outcomes.

This study has several limitations. First, it was a retrospective and single institutional study, and the number of patients was small because of the low incidence of the condition described. Multivariate analysis should assess which factors are independent and contribute significantly to the success of the TAE, however could not be performed due to the small number of patients. Further studies with a large number of patients are required to confirm the results. Second, embolization was performed by multiple interventional radiologists in our institute. The embolization method was not completely standardized, particularly in the choice of embolic materials. However, in each case, multiple skilled interventional radiologists performed embolization, so the quality of the procedure was preserved. Third, as mentioned above, long-term follow-up data were lacking in our study because most patients were referred from other hospitals. Although there were no major clinical complications during their hospital stay in our institution, follow-up after discharge and transfer was done in other hospitals, and not all patients were assessed for a long period.

In conclusion, our findings suggest that S-TAE is an effective and safe treatment for intractable PPH due to GTT after vaginal delivery. Pre-procedural CE-CT may be useful for detecting the culprit artery and be helpful in performing S-TAE.
